# Efficacy and safety of nasal high-flow oxygen in COPD patients

**DOI:** 10.1186/s12890-017-0486-3

**Published:** 2017-11-17

**Authors:** Helene Vogelsinger, Michael Halank, Silke Braun, Heinrike Wilkens, Thomas Geiser, Sebastian Ott, Armin Stucki, Christian M. Kaehler

**Affiliations:** 1 Pneumology, Internal Medicine II, Department of Internal Medicine, Medical University of Innsbruck, Innsbruck, Austria; 2 Pneumology, Medical Clinic and Polyclinic, University Hospital Carl Gustav Carus, Dresden, Germany; 3 Pneumology, Saarland University Medical Centre, Homburg, Germany; 4 Department for Pulmonary Medicine, University Hospital Inselspital, Bern, Switzerland; 5 Pneumology, Berner REHA Zentrum Heiligenschwendi, Heiligenschwendi, Switzerland; 6 Pneumology, Lung Centre Southwest, Wangen im Allgäu, Germany

**Keywords:** High-flow oxygen, short-term nasal highflow., COPD., normocapnic., hypercapnic.

## Abstract

**Background:**

Nasal high-flow oxygen therapy (HFOT) is a novel treatment option for patients suffering from acute or chronic respiratory failure. Aim of our study was to compare safety and efficacy of HFOT with those of conventional oxygen treatment (COT) in normo- and hypercapnic COPD patients.

**Methods:**

A single cohort of 77 clinically stable hypoxemic patients with an indication for long-term oxygen treatment (LTOT) with or without hypercapnia successively received COT and HFOT for 60 min each, including oxygen adaption and separated by a 30 min washout phase.

**Results:**

HFOT was well-tolerated in all patients. A significant decrease in PaCO_2_ was observed during oxygen adaption of HFOT, and increased PaO_2_ coincided with significantly increased SpO_2_ and decreased AaDO_2_ during both treatment phases. Even at a flow rate of 15 L/min, oxygen requirement delivered as air mixture by HFOT tended to be lower than that of COT (2.2 L/min). Not only was no increase in static or dynamic lung volumes observed during HFOT, but even was a significant reduction of residual lung volume measured in 36 patients (28%).

**Conclusions:**

Thus, short-term use of HFOT is safe in both normocapnic and hypercapnic COPD patients. Lower oxygen levels were effective in correcting hypoxemic respiratory failure and reducing hypercapnia, leading to a reduced amount of oxygen consumption. Long-term studies are needed to assess safety, tolerability, and clinical efficacy of HFOT.

**Trial registration:**

ClinicalTrials.gov NCT01686893 13.09.2012 retrospectively registered (STIT-1) and NCT01693146 14.09.2012 retrospectively registered (STIT-2). Studies were approved by the local ethics committee (Ethikkommission der Medizinischen Universität Innsbruck, Studienkennzahl UN3547, Sitzungsnummer 274/4.19).

## Summary

Short-term nasal highflow oxygen therapy (HFOT) is safe and efficacious in normocapnic and hypercapnic COPD patients.

## Background

Nasal high-flow oxygen therapy (HFOT) is a novel non-invasive alternative to conventional oxygen treatment (COT) and an alternative to non-invasive ventilation in selected patients. It is based on the transnasal application of a preheated and moistened air-oxygen composition at high flow rates [1, 2]. Besides pharmaceutical treatment options, long-term oxygen treatment (LTOT) and non-invasive ventilation have become a main emphasis in the therapy of respiratory insufficiency including chronic obstructive pulmonary disease (COPD), sleep apnoea, pulmonary oedema, and other conditions [3]. The prevalence of COPD worldwide is between 9 and 10% in people over the age of 40, with the incidence significantly varying between countries, i.e. ranging from 0.2% in Japan to 37% in the USA. Men are more often affected than women [4, 5]. According to the WHO, COPD is the fourth leading cause of death and is supposed to become the third leading cause by 2020 [6]. COPD is marked by an airflow limitation with persistent and progressive courses of breathlessness, frequently associated with chronic productive cough and chest tightness [7].

As a conventional treatment option, typically applied in case of persistent hypoxaemia (PaO_2_ < 55 mmHg or <60 mmHg upon signs of right heart failure or polycythaemia), LTOT via a nasal cannula or oxygen mask has been successfully performed for decades. So far, LTOT represents the only life-extending therapy in severe lung disease [8, 9]. However, long-term modes of treatment remain inapplicable in patients with restricting factors and usually lead to limitations in the patients’ mobility and quality of life [10]. Recent studies illustrated further limitations of conventional LTOT, becoming particularly evident in cases of exacerbations or at later stages of the disease [11]. As an optional way of treatment that recently moved into focus, HFOT has been investigated, and specific devices such as TNI®20 oxy (TNI medical AG, Würzburg, Germany) have been developed [12]. For HFOT, humidification and preheating of the applied gas offers a suitable precondition to achieve high flow rates and prevent airway dehydration. An obvious advantage of nasal application over respiratory masks lies in improved ways of daily activity, free ability to communicate, and higher levels of compliance [13].

Successful applications of HFOT in the past included paediatric treatments, in which intubation or continuous positive airway pressure could be prevented, and the treatment of respiratory distress syndrome [14]. Similarly, treatment approaches in premature infants have been shown to exert beneficial effects in cases of apnoea and other indications in children, such as infectious bronchiolitis, pneumonia, and congestive heart failure [14, 15]. A number of recent clinical investigations provided a strong input to the HFOT strategy and underlined the benefits of various HFOT applications [16–20].

So far, however, clear information is limited on whether HFOT is reliably beneficial for the broader treatment of respiratory insufficiencies and may provide advantages over conventional forms of therapy. In this study, the question was addressed whether HFOT provides the same safety and efficacy as COT in the treatment of hypoxaemic COPD patients with both normo- or hypercapnia. Primary and secondary endpoints of the study were set as changes in the partial oxygen and carbon dioxide pressure (PaO_2_, PaCO_2_), the alveolar to arterial oxygen pressure difference (AaDO_2_), and peripheral oxygen saturation (SpO_2_) at a defined oxygen flow rate, as well as safety of HFOT as assessed by lung volume changes. Thus, our study verifies for the first time that HFOT is safe and efficacious in COPD patients with or without hypercapnia.

## Methods

### Study population

The study population comprised a single cohort of 77 COPD patients, who were already treated by conventional LTOT and stabilized, then awaiting a potential inclusion into the subsequent HFOT studies STIT-1 and -2 (Short Time TNI Treatment). Inclusion criteria of the STIT-1 study on normocapnic COPD patients were defined by the indication for LTOT (PaO_2_ < 55 mmHg or <60 mmHg upon signs of right heart failure or polycythaemia), age 30–80 years, and functional class COPD GOLD IV (as defined by FEV_1_/FVC < 70%, post-bronchodilator forced expiratory volume in 1 s [FEV_1_] < 30% pred., or <50% pred. With LTOT indication). Two groups were defined by the post-bronchodilator FEV_1_, with group 1 corresponding to 30% pred. ≤ FEV_1_ < 50% pred. And group 2 corresponding to FEV_1_ < 30% pred. [21]. The number of patients announced/analysed was 50/50 in STIT-1. Inclusion criteria of the STIT-2 study on hypercapnic COPD patients were an indication for LTOT (see above), PaCO_2_ > 45 mmHg at rest without oxygen supplementation, age 30–85 years, and functional class COPD GOLD IV (see above). The number of patients announced/analysed was 20/27. Exclusion criteria in both studies were clinical instability, lack of option for testing lung function, exacerbation within the last 14 days, serious concomitant diseases, severe anaemia (haemoglobin <8.5 g/L), missing consent or participation in any other ongoing study. Any oral or inhaled medication approved for COPD therapy was allowed. Studies STIT-1 and STIT-2 lasted from 02/08 and 11/11 (first patient first visit) to 12/11 and 06/12, respectively. Both studies were extended to 06/14. Studies were registered at ClinicalTrials.gov NCT01686893 (STIT-1 [22]) and NCT01693146 (STIT-2 [23]) and approved by the local ethics committee.

### Study design

This prospective study was directed to a single cohort of patients participating in sub study STIT-1 or STIT-2. During the screening visit, arranged 2 weeks prior to start of each sub study, informed consent was obtained. Every patient was informed about the new method of HFOT and its possible effects for COPD patients, furthermore about the course of the study and the extra investigations during the study. The patient’s history as well as demographic and clinical parameters were assessed. Patients (*n* = 77) received first COT, which was followed by HFOT, each for 60 min including initial oxygen adaption (see below). For COT, oxygen was applied using the hospital’s standard oxygen system and standard nasal prongs, and TNI®20 oxy (TNI medical AG, Würzburg, Germany) was employed as HFOT device. Both treatment phases were separated by a 30 min washout phase in a sitting position, without oxygen (see below). Physical examinations, analysis of vital signs, and blood gas analysis were performed repeatedly as follows: at the screening visit, at baseline, during both oxygen adaption phases, after the 30 min washout phase, and after COT and HFOT treatment. Bodyplethysmography, spirometry and DLCO (diffusing capacity of lung carbon monoxide) measurement were performed at the screening visit, at baseline and after COT and HFOT. Primary endpoint was the change in PaO_2_ in the arterialised capillary blood (blood gas analysis) at a defined oxygen flow rate (L/min). Secondary endpoints were changes in SpO_2_, PaCO_2_ and AaDO_2_ in the arterialised capillary blood (blood gas analysis) at a defined oxygen flow rate (L/min), and safety of the device in normocapnic (STIT-1) and hypercapnic COPD patients (STIT-2), as defined as no increase in the residual volume (RV) and the total lung capacity (TLC) > 15% of the mean actual value. After treatment, patient satisfaction was assessed in non-standardized surveys.

### Oxygen adaptation of COT and HFOT

Oxygen adaption of both COT and HFOT followed the same standard protocol. Nasal oxygen insufflation with conventional oxygen started at 0.5 L/min and was increased in steps of 0.5 to 1 L/min until PaO_2_ was >60 mmHg or PaO_2_ increased by ≥10 mmHg as compared to the initial value. After successful adaptation, patients received nasal oxygen insufflation at the established flow rate for the remaining duration of the hour to be completed. Blood gas analysis, performed after 10 min at a defined flow rate, was mandatory at the beginning of oxygen adaptation and was completed 1 h thereafter. Oxygen flow rate could be increased without blood gas analysis if a SpO_2_ > 90% was not reached. A 30 min washout phase at rest, in a sitting position, without oxygen then followed COT. During the washout phase, PaO_2_ should decline to initial levels (± 2 mmHg). Adaptation to the HFOT phase was achieved by titration starting from room air conditions (oxygen admixture 0 L/min) towards a nasal oxygen insufflation of 15 L/min, under oxygen admixture steps of 0.5 L/min until reaching a value of PaO_2_ > 60 mmHg or an increase of ≥10 mmHg (in contrast, adaptation to COT started at 0.5 L/min). All subsequent steps followed the protocol described above.

### Procedures

For clinical examinations, vital signs (blood pressure and heart rate) were measured at the beginning and at the end of each point of the study. Blood gases were taken from the hyperaemic ear lobe (anointed with Finalgon [Boehringer-Ingelheim] to increase perfusion) with a single-use system. Hyperaemisation leads to arterialisation of capillary blood, thus the arterial values (PaO_2_, PaCO_2_) are reflected adequately. The collected blood samples were analysed with a routinely used and daily calibrated blood gas analysis device within 2 min after puncture. The bodyplethysmography, spirometry and DLCO measurements comprised the assessment of dynamic and static lung parameters as follows: (i) dynamic volumes such as FEV_1_% (forced expiratory volume in 1 s), RAW (airway resistance) and (ii) static volumes such as ERV (expiratory reserve volume), IC (inspiratory capacity), VC (vital capacity, Vcex and Vcin), RV (residual volume), and TLC (total lung capacity). Static and dynamic lung volumes were recorded with the MasterScreen Body© (CareFusion, Hoechberg, Germany) and Pneumotach© (Jaeger, Würzburg, Germany) providing flow measurements in a range up to 20 L/s and an accuracy of ±2%. Volume determination was performed by digital integration within a range of ±20 L and an accuracy of ±3% or ±50 ml. Pressure measurements were piezoresistive with an accuracy of ±2%.

### Statistic evaluation

For statistical analyses, continuous data were described in terms of number, minimum, maximum, mean value, median, and standard deviation. Discrete data sets were described in terms of number and percentage. To investigate differences in oxygen quantities, a paired sample test was used. As the central issue of statistical evaluation, the oxygen flow required to achieve a PaO_2_ of >60 mmHg or an increase by at least 10 mmHg when using COT were contrasted to HFOT. The software used was SPSS Statistics 15.0. All values are given as mean ± SD, and data were considered as significant by the Student’s t-test when *p* < 0.001.

## Results

### Patient characteristics

**Fig. 1:**
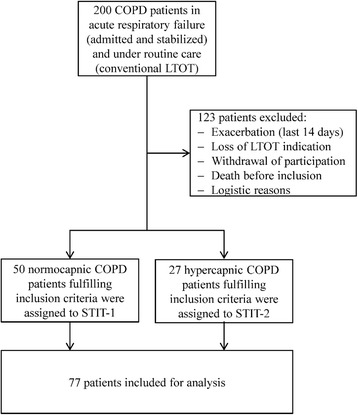
Enrollment of normocapnic and hypercapnic COPD patients in STIT-1 and STIT-2. 200 patients with COPD GOLD IV were screened for study inclusion. The final study population comprised a single cohort of 77 stable COPD patients, who were already treated by conventional long term oxygen treatment (LTOT), then awaiting a potential inclusion into the subsequent HFOT studies STIT-1 and -2 (Short Time TNI Treatment)

Characteristics of the study population, based on defined inclusion and exclusion criteria, is depicted in Fig. 1. Patients of both sub studies were, on average, 66.2 ± 8.5 years old (range 47–84 years), and males were predominant (74%). Their mean FEV_1_ was 31.0 ± 11.4% of the predicted value, and patients were classified into FEV_1_ 30–50% pred. (*n* = 38) and FEV_1_ < 30% pred. (*n* = 39). At screening visit, mean (± SD) PaO_2_ and PaCO_2_ values were 49.6 ± 6.2 mmHg and 43.2 ± 6.3 mmHg, respectively, with a SpO_2_ at 91 ± 2%. Mean LTOT oxygen flow rates (± SD) of 2.2 ± 1.6 L/min (anamnestic) indicated that patients were clinically stable (table 1).

**Table 1:** Patient characteristics and lung function at screening visit

	STIT-1	STIT-2	STIT-1 + 2
Patient demographics			
Sample size (n)	50	27	77
Sex (male/female)	42/8	15/12	57/20
Age, years (mean ± SD)	67.2 ± 8.5	64.4 ± 8.2	66.2 ± 8.5
FEV_1_ group: 30–50% (n)	31	7	38
FEV_1_ group: < 30% (n)	19	20	39
Lung function			
pred. FEV_1_, % (mean ± SD)	34.5 ± 11.2	24.9 ± 9.2	31.0 ± 11.4
LTOT, O_2_ L/min (mean ± SD)	2.2 ± 1.7	2.4 ± 1.4	2.2 ± 1.6
PaO_2_, mmHg (mean ± SD)	51.0 ± 6.1	47.0 ± 5.6	49.6 ± 6.2
PaCO_2_, mmHg (mean ± SD)	40.0 ± 4.1	49.1 ± 5.4	43.2 ± 6.3
SpO_2_ (%)	91 ± 2	89 ± 3	91 ± 2

FEV_1_: forced expiratory volume in 1 s; LTOT: long term oxygen therapy

### Blood gas analysis and oxygen requirement

**Fig. 2:**
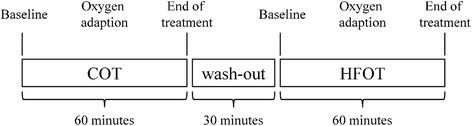
Study procedures and measurements. Stable patients with COPD GOLD IV (*n* = 77) successively received conventional oxygen therapy (COT) and nasal high-flow oxygen therapy (HFOT), each for 60 min and separated by a 30 min washout phase. Blood gas and vital signs were analysed at the screening visit, at baseline, after oxygen adaption (PaO_2_ > 60 mmHg or increased by ≥10 mmHg when compared to baseline), and at the end of each treatment. Bodyplethysmography, spirometry and DLCO measurements were performed at the screening visit, at baseline, and at the end of each 1-h treatment session

Patients (*n* = 77) successively received COT and HFOT, each for 60 min and separated by a 30 min washout phase (Fig. 2). Blood gas and vital signs were analysed at the screening visit, at baseline, after oxygen adaption (PaO_2_ > 60 mmHg or increased by ≥10 mmHg when compared to baseline), and at the end of each treatment. Bodyplethysmography, spirometry and DLCO measurements were performed at the screening visit, at baseline, and at the end of each 1-h treatment session.

**Fig. 3:**
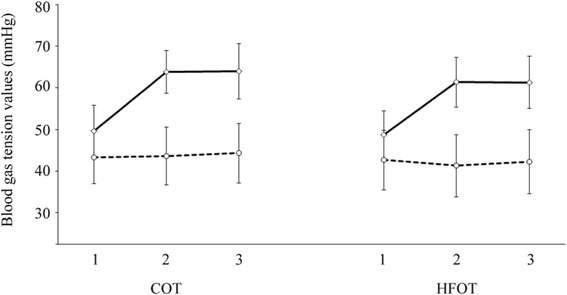
Mean blood gas values during conventional oxygen therapy (COT) and nasal high-flow oxygen therapy (HFOT). Blood samples (n = 77) were subjected to measurements of PaCO_2_ (dotted line) and PaO_2_ (solid line) at baseline (1), after 10–60 min of oxygen adaptation until reaching a value of PaO_2_ > 60 mmHg or an increase of ≥10 mmHg (2), and at the end of the 60-min treatment period [including adaption] (3)

The starting mean baseline PaO_2_ of 49.6 ± 6.2 mmHg and 48.7 ± 5.8 mmHg increased to 63.8 ± 5.1 mmHg and 61.4 ± 6.0 mmHg during oxygen adaption of COT and HFOT, respectively, and remained constant during the remaining hour of treatment (Fig. 3).

After both treatments, SpO_2_ baseline levels were significantly increased (*p* < 0.0001; data not shown). HFOT reduced PaCO_2_ levels already during oxygen adaptation (−2.36 mmHg, *p* < 0.038) which remained on a lower level until the end of treatment (−2.11 mmHg, *p* < 0.077). In contrast, PaCO_2_ slightly increased during COT (Fig. 3), and PaCO_2_ differences between COT and HFOT were significant after both oxygen adaptation and 1-h treatment session (*p* < 0.0001). Under these HFOT conditions (constant flow rate of 15 L/min), overall oxygen requirement of HFOT was lower than that of COT in normocapnic (1.87 ± 1.57 L/min vs. 2.07 ± 1.65 L/min) and hypercapnic (2.09 ± 1.14 L/min vs. 2.15 ± 1.37 L/min,) COPD patients.

**Fig. 4:**
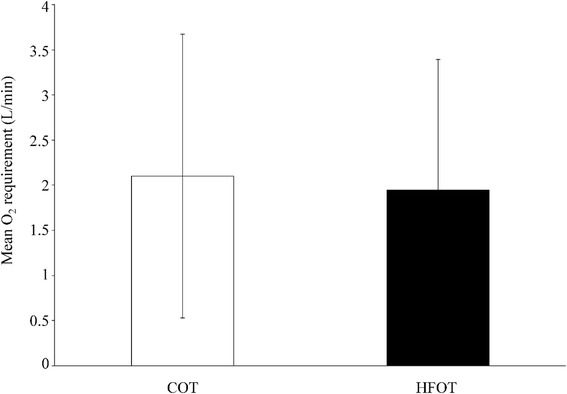
Oxygen requirement during nasal high-flow oxygen therapy (HFOT). Mean oxygen consumption during conventional oxygen therapy (COT) and HFOT was recorded in 77 patients with stable COPD GOLD IV(combining 50 normocapnic and 27 hypercapnic COPD patients), as assessed by blood gas analysis

Figure 4 shows the combined data for both normocapnic and hypercapnic patients, demonstrating the reduced amount of oxygen required to achieve comparable oxygenation during HFOT (1.95 ± 1.45 L/min vs. 2.1 ± 1.57 L/min). A FiO_2_ of 31.11 ± 7.63% during HFOT was documented to achieve required patients´ oxygenation. Of note, in some cases (*n* = 6), in which the patient’s need of oxygen remained moderately low, room air concentrations were sufficient for HFOT without any oxygen admixture. In addition, AaDO_2_ declined from 50.33 to 35.87 mmHg under COT and from 51.91 to 39.49 mmHg under HFOT, respectively, with each decrease being significant (*p* < 0.0001).

### Safety

**Fig. 5:**
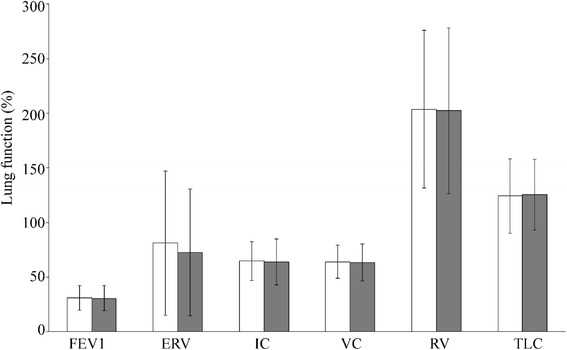
Determination of lung function parameters under nasal high-flow oxygen therapy (HFOT) in 77 stable COPD patients. Dynamic and static lung parameters after both conventional oxygen therapy (COT; white bars) and HFOT (grey bars) were expressed in percent of the predicted value: FEV1 (forced expiratory volume in 1 s), ERV (expiratory reserve volume), IC (inspiratory capacity), VC (vital capacity), RV (residual volume), and TLC (total lung capacity)

No alterations of lung function parameters were detected during the study period (Fig. 5). Interestingly, in 28% of patients (36/77), the RV was significantly lower after HFOT than that after COT (4.17 ± 1.03 L vs. 4.68 ± 1.53 L; *p* < 0.0001). In addition, there were no significant differences in DLCO (3.96 ± 1.59 mmol/min/kPa vs. 3.89 ± 1.51 mmol/min/kPa) and RAW after both treatment regimens (0.83 ± 0.84 kPa/L/s vs. 0.87 ± 0.57 kPa/L/s). No adverse events and no significant alterations in blood pressure or cardiac frequency were noted. HFOT was well tolerated by all 77 patients.

## Discussion

In this study, we analysed HFOT as a novel treatment option for chronic hypoxaemic respiratory failure in both normocapnic and hypercapnic COPD patients, and compared it to the classical mode of COT concerning efficacy of oxygenation and safety. The main results of the study were that (i) HFOT was well-tolerated by all 77 patients, (ii) the level of oxygen requirement (FiO2) was generally lower under HFOT, (iii) hypercapnia was significantly reduced under HFOT, and (iv) HFOT was safe as no increase in residual volumes or any alteration of total lung volumes occurred. We therefore conclude that HFOT offers therapeutic benefits for both normocapnic and hypercapnic COPD patients as a new procedure for the future. As compared to standard therapy (COT), lower levels of oxygen were effective in correcting hypoxaemic respiratory failure and reducing hypercapnia, ultimately leading to an economization of oxygen consumption.

To date, COT has been the most frequently used oxygenation assistance (LTOT) in the therapy of severe COPD that was shown to improve survival. With HFOT, a completely new method has recently been introduced. However, data on safety in severely ill COPD patients with hypoxaemia, hyperinflation and consequently hypercapnia are still missing. Therefore, the aim of our study was to examine general parameters of reliability and usefulness in the treatment of severe COPD. Under HFOT, PaO_2_ substantially increased and remained constant throughout the period of treatment. Likewise, SpO_2_ was significantly increased, while PaCO_2_ levels already declined during oxygen adaptation of HFOT. Furthermore, also AaDO_2_ was significantly reduced by both COT and HFOT.

Regarding oxygenation, HFOT seems to be superior to COT. In our experiments lower FiO2 rates were necessary to achieve the predefined PaO_2_ in our patients. Utilizing COT 100% of oxygen is inspired. Wettstein et al. reported that using COT FiO2 increases with increasing flow rates. COT was delivered at a mean flow rate of 2.2 ± 1.6 L/min. In the data published by Wettstein and colleagues a pharyngeal FiO2 of 0.30 to 0.38 can be measured at a COT flow rate of 2 L/min. However, also FiO_2_ of 31.11 ± 7.63% during HFOT was measured with higher flow rates and applying an air/oxygen mixture. Higher flow rates might even further reduce oxygen demand in these patients, which might be of socio-economical relevance.

Thus, one of the essential findings was the clear reduction of PaCO_2_ during HFOT compared to COT as verified in both subgroups of the patient collective. As a potential reason, an improved washout effect of the nasopharyngeal dead space or, alternatively, an increase in pharyngeal pressure due to the elevated flow rate under HFOT appears plausible [14]. Hereby, the decrease of PaCO_2_ in hypercapnic patients needs to be stressed as a quality of particular importance since it lowers the risk of respiratory arrest, especially in cases of acute exacerbation. This finding is supported by previous studies showing a significant decline in PaCO_2_ with HFOT [12, 24]. Frizzoli et al. [24] observed decreasing PaCO_2_ with rising flow and explained the effect with improved wash out efficiency of the nasopharyngeal dead space. According to these results, PaCO_2_ should not be affected by tracheal pressure but solely by higher flow rates. This possibility, however, was contradicted by Mc Ginley et al. [13], explaining this HFOT-based effect by an increase in pharyngeal pressure. In essence, these findings point to the hypothesis of an overall improvement in oxygenation by HFOT, which has not been described in the literature until now [1, 3, 25].

As far as safety parameters are concerned, it should be stressed that during HFOT neither deterioration of lung volumes, nor significant differences in DLCO, RAW levels or other adverse events were noted. The high level of patient satisfaction was deemed as a further success of the use of HFOT in the cohort. All patients rated HFOT as ‘pleasant’, which is compatible with earlier studies stressing the improved comfort as based on low levels of dyspnoea and mouth dryness as well as a lack of restrictions in food ingestion or speaking [26]. This positive general evaluation was also supported by other observations, such as a reduction of dyspnoea attributed to a correction of hypoxaemia and a reduction of the respiratory rate and the humidification providing a higher secretion clearance and an improved mucociliary function in the airways [26].

Recently, the benefit of HFOT applications has been demonstrated by several reports, underlining that HFOT is well tolerated by patients with mild to moderate hypoxic respiratory failure [19]. Frat et al. [16] demonstrated improved survival rates among HFOT treated patients with acute hypoxaemic respiratory failure when compared to patients treated by COT or non-invasive ventilation. In patients with acute respiratory failure, the coordination of breathing-related movements of the rib cage and abdominal wall is often impaired, leading to respiratory muscle fatigue [27]. This thoraco-abdominal impairment of synchrony could be improved by HFOT in patients with mild to moderate respiratory failure [17]. Moreover, in patients with post-extubation respiratory failure, HFOT was as effective as non-invasive ventilation in avoiding reintubation of patients [18, 20].

Limitations of our study might be seen in the relatively small overall patient number, so that some constraints in delivering representative data similarly valuable for greater collectives cannot be fully excluded. In addition, the short-term regimen reduced to a single one-hour treatment might render some room for optimization, so that a third trial under varied conditions is presently in the planning phase (termed STIT-3 conceived as a long-term study). Another minor point of limitation might have been given by the uniform application of the single low flow rate of 15 L/min, which might possibly be even further optimised in future studies as currently flow rates up to 50 L/min are reported to be used.

Overall, the study outcome is considered very promising leading to the following conclusions. We suggest HFOT as a novel option of non-invasive treatment providing an efficacious and safe mode of oxygenation for COPD patients. Parameters being at least comparable or even more advantageous compared to COT included the lower level of oxygen requirement, the significant decrease in PaCO_2_, the conservation of lung functionality, and a high level of patient satisfaction. A reduction of oxygen requirement was seen in both patient groups, with an even more pronounced effect in normocapnic than in hypercapnic patients. Combined, the findings demonstrate various aspects of beneficial effects for both normo- and hypercapnic COPD patients using the novel HFOT regimen so that larger studies in the near future might expand on this set of data.

## Conclusions

Short-term use of HFOT is safe in normocapnic and hypercapnic COPD patients. During HFOT lower oxygen levels were effective in correcting hypoxemic respiratory failure and reducing hypercapnia, leading to a reduced amount of oxygen consumption.

## Abbreviations

AaDO_2_: Alveolar to arterial oxygen pressure difference
COPD: Chronic obstructive pulmonary disease
COT: Conventional oxygen treatment
DLCO: Diffusing capacity of lung carbon monoxide
ERV: Expiratory reserve volume
FEV_1_: Forced expiratory volume in one second
FiO2: Fraction of inspired oxygen
FVC: Forced vital capacity
GOLD IV: Global Initiative for Chronic Obstructive Lung Disease IV
HFOT: Highflow oxygen therapy
IC: Inspiratory capacity
LTOT: Long-term oxygen treatment
PaCO_2_: Partial oxygen pressure (arterialised)
PaO_2_: Partial carbon dioxide pressure (arterialised)
RAW: Airway resistance
RV: Residual volume
SD: Standard deviation
SpO_2_: Peripheral oxygen saturation
STIT-1: Short time TNI treatment 1 (normocapnic COPD patients)
STIT-2: Short time TNI treatment 2 (hypercapnic COPD patients)
STIT-3: Long time TNI treatment
TLC: Total lung capacity
TNI: Transnasal Insufflation
VC: Vital capacity
Vcex: Exspiratory vital capacity
Vcin: Inspiratory vital capacity
WHO: World Health Organization
